# Nasogastric Tube Syndrome Treated by Replacement With a Smaller-Diameter Tube: A Case Report

**DOI:** 10.7759/cureus.78202

**Published:** 2025-01-29

**Authors:** Kazuya Hara, Takashi Shigematsu, Hiroki Ikeda, Ken Iwanaga, Ichiro Fujishima

**Affiliations:** 1 Department of Rehabilitation Medicine, Hamamatsu City Rehabilitation Hospital, Hamamatsu, JPN; 2 Department of Otolaryngology, Head and Neck Surgery, Graduate School of Medicine, Kyoto University, Kyoto, JPN

**Keywords:** lateral medullary syndrome (wallenberg syndrome), nasogastric tube syndrome, post-stroke dysphagia, post-tracheostomy, smaller-diameter tube

## Abstract

Nasogastric tube syndrome (NGTS) is a severe and potentially life-threatening complication of the nasogastric tube. NGTS is characterized by throat pain and abductor dysfunction of vocal cords due to the presence of the NGT. NGTS sometimes progresses to acute upper airway obstruction caused by vocal cord paralysis or laryngeal infection. Early recognition and appropriate management are essential to prevent serious outcomes. Removing the nasogastric tube is generally recommended for treatment. Here, we present a case of NGTS, wherein the symptoms were improved by replacing the nasogastric tube with a smaller-diameter tube rather than removing it entirely. A 53-year-old woman suffering from left lateral medullary syndrome with a tracheostomy was admitted for dysphagia rehabilitation. She developed NGTS because of the presence of a 12-Fr nasogastric tube. Videoendoscopic evaluation of swallowing revealed right vocal cord abduction paralysis and bilateral arytenoid edema. Her oral intake was restricted because of lateral medullary syndrome and NGTS. As she was not at risk for upper airway obstruction from NGTS because of the tracheostomy, we replaced the nasogastric tube with a smaller-diameter (8-Fr) tube, resulting in an improvement in her dysphagia and NGTS. Considering that NGTS can make dysphagia worse, replacing a nasogastric tube with a smaller-diameter (8-Fr) tube may be an effective treatment for NGTS. Although this approach may extend the overall treatment duration, it offers a feasible option for patients requiring continued nasogastric tube feeding without removing the tube.

## Introduction

Nasogastric tube syndrome (NGTS), a rare but potentially life-threatening condition characterized by upper airway obstruction, is typically defined by the presence of a nasogastric tube, arytenoid edema, and either bilateral or unilateral vocal cord paralysis or abductor dysfunction [1]. In severe cases, patients may develop significant respiratory distress, requiring emergency tracheostomy to secure the airway. Removing the nasogastric tube is generally considered the most effective treatment for NGTS [2].

Previous studies have suggested that softer, smaller-diameter tubes may help prevent NGTS [3]. However, no documented cases exist where NGTS was successfully treated solely by replacing the existing tube with a smaller-diameter one. Additionally, while NGTS is known to cause dysphagia, there are no reported cases in the literature describing NGTS-induced dysphagia severe enough to restrict oral intake specifically.

This report presents a case of NGTS with dysphagia that was improved by replacing the 12-Fr nasogastric tube with a smaller-diameter (8-Fr) tube.

## Case presentation

A 53-year-old woman was transferred to our hospital for dysphagia rehabilitation. Three months before the transfer, she developed dizziness and posterior cervical pain and was admitted to a general hospital. MRI revealed that the patient had left lateral medullary and cerebellar infarction caused by a left vertebral artery dissection. On the following day, the patient developed a decreased level of consciousness due to hydrocephalus, requiring emergency ventricular drainage surgery and intensive care unit admission. A tracheostomy was performed on the sixth day of hospitalization to secure her airway. Although her consciousness disorder improved, she remained unable to take anything orally because of dysphagia and depended on nasogastric tube feeding for all meals. Three months after the initial symptom onset, she was transferred to our hospital for dysphagia rehabilitation.

**Figure 1:**
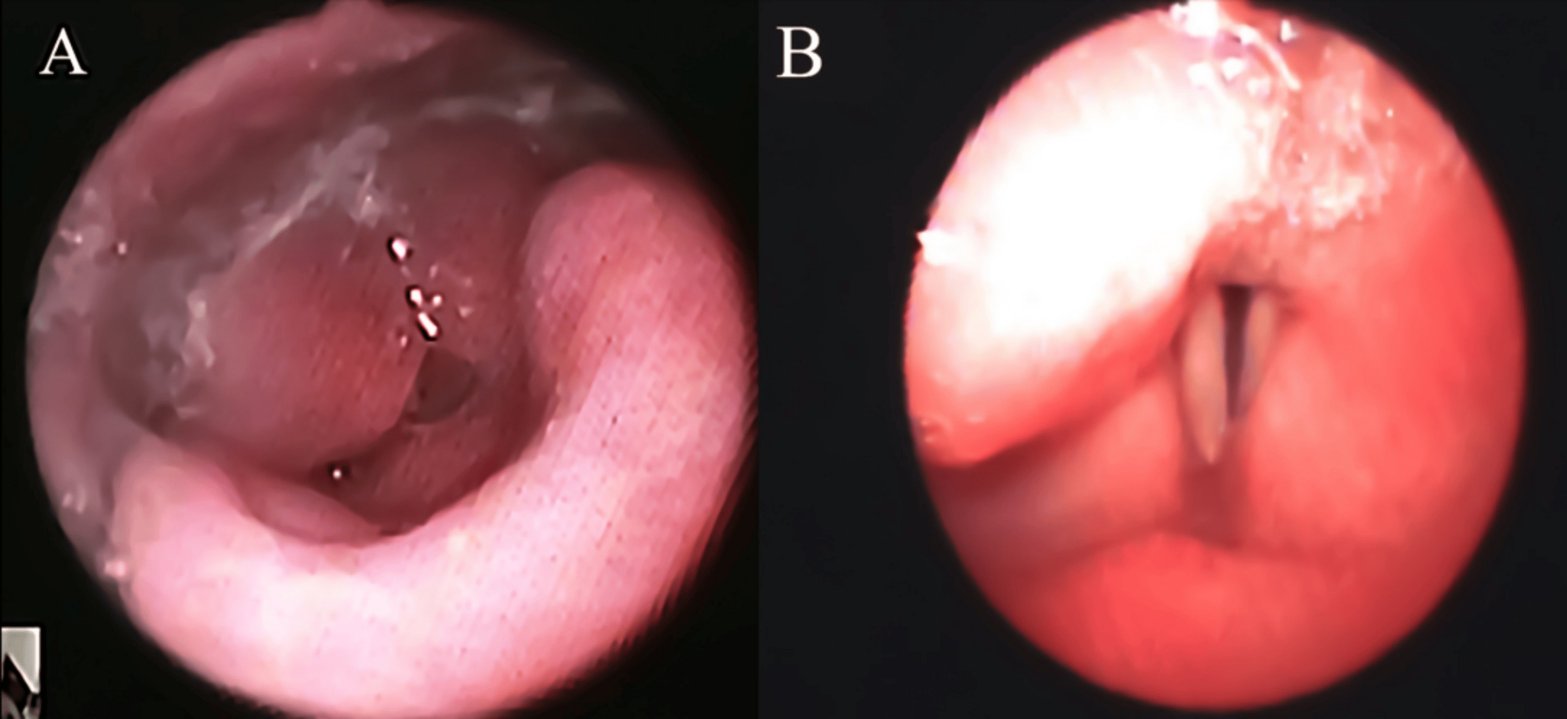
Videoendoscopic evaluation of swallowing showing (A) bilateral arytenoid edema and (B) left vocal cord paralysis and right vocal cord abduction paralysis

Her symptoms were left ptosis, pupil constriction, and ataxia in the left hemisphere, consistent with a lateral medullary syndrome, without any evident sensory deficits. Clinical evaluation of dysphagia revealed a normal repetitive saliva swallowing test with two swallows in 30 seconds and a Mann Assessment of Swallowing Ability score of 88 [4], indicating severe dysphagia and severe aspiration risk. Food Intake Level Scale (FILS) score was level 2 [5], indicating that the patient was only capable of foodless swallowing exercises (indirect therapy). Considering that the patient was obese and had thick subcutaneous tissue in the neck, an adjustable tracheal cannula (Fuji Systems Corporation, Adjust Fit®) was inserted. Videoendoscopic evaluation of swallowing (VE) was conducted on the third day of hospitalization and revealed left vocal cord paralysis and saliva pooling in the left piriform sinus, which were both attributed to the left lateral medullary syndrome. A 12-Fr nasogastric tube passed from the right nasal cavity to the center of the bilateral piriform sinuses, accompanied by right vocal cord abduction paralysis and bilateral arytenoid edema (Figure 1).

**Figure 2:**
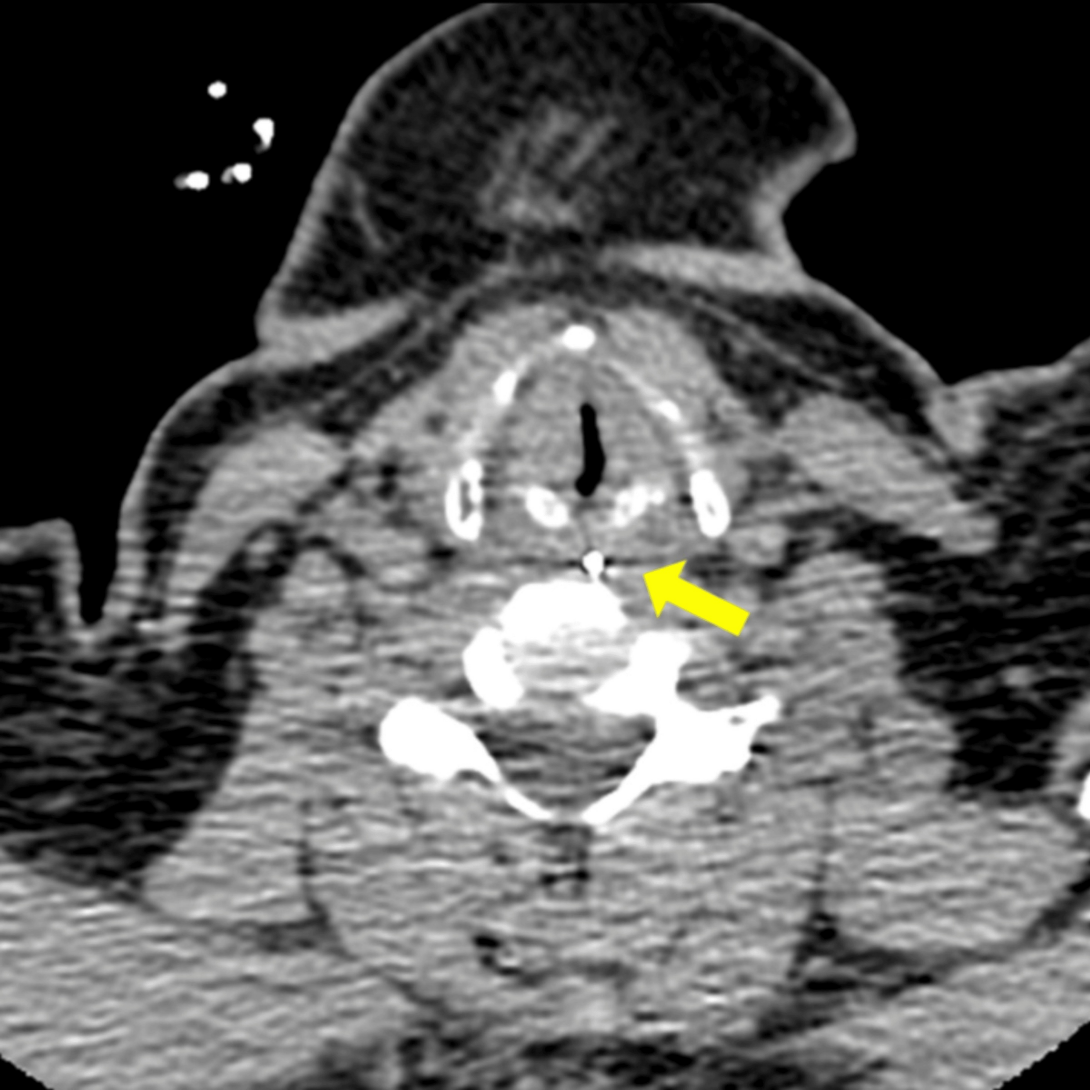
CT showing the nasogastric tube passing almost midway through the bilateral piriform sinuses (yellow arrow)

Cervical CT revealed the nasogastric tube positioned near the midline of the piriform sinus and no structural abnormalities causing recurrent nerve palsy (Figure 2).

Considering the bilateral vocal cord paresis associated with nasogastric tube insertion and the absence of other causative factors, the patient was diagnosed with NGTS. Videofluoroscopic examination of swallowing (VF) revealed reduced pharyngeal contraction and limited opening of the bilateral upper esophageal sphincter, which made it difficult for the patient to ingest even gelatin jelly orally.

In this case, we aimed to transition from nasogastric tube feeding to intermittent oral-esophageal tube feeding and balloon dilation therapy. However, we could not use this approach because of her strong gag reflex. Therefore, we replaced the currently inserted nasogastric tube (Cliny®, 12 Fr, Create Medic Co.) with a smaller-diameter tube (J-feed®, 8-Fr, JMS Corporation). We could not pass the nasogastric tube through the left piriform fossa, so we inserted the tube from the right nasal cavity to the right piriform fossa. The tracheal cannula was also changed from an adjustable cannula to a cuff-button-like cannula with a speaking valve (Tracheal Stoma Retainer® 11 mm × 32 mm, Koken Corporation). To enhance her oral intake, we implemented a rehabilitation program that included neuromuscular electrical stimulation therapy using VitalStim® (InterReha Co. Ltd., Tokyo, Japan) [6], forehead exercises for suprahyoid muscles [7], and lingual resistance exercises.

**Figure 3:**
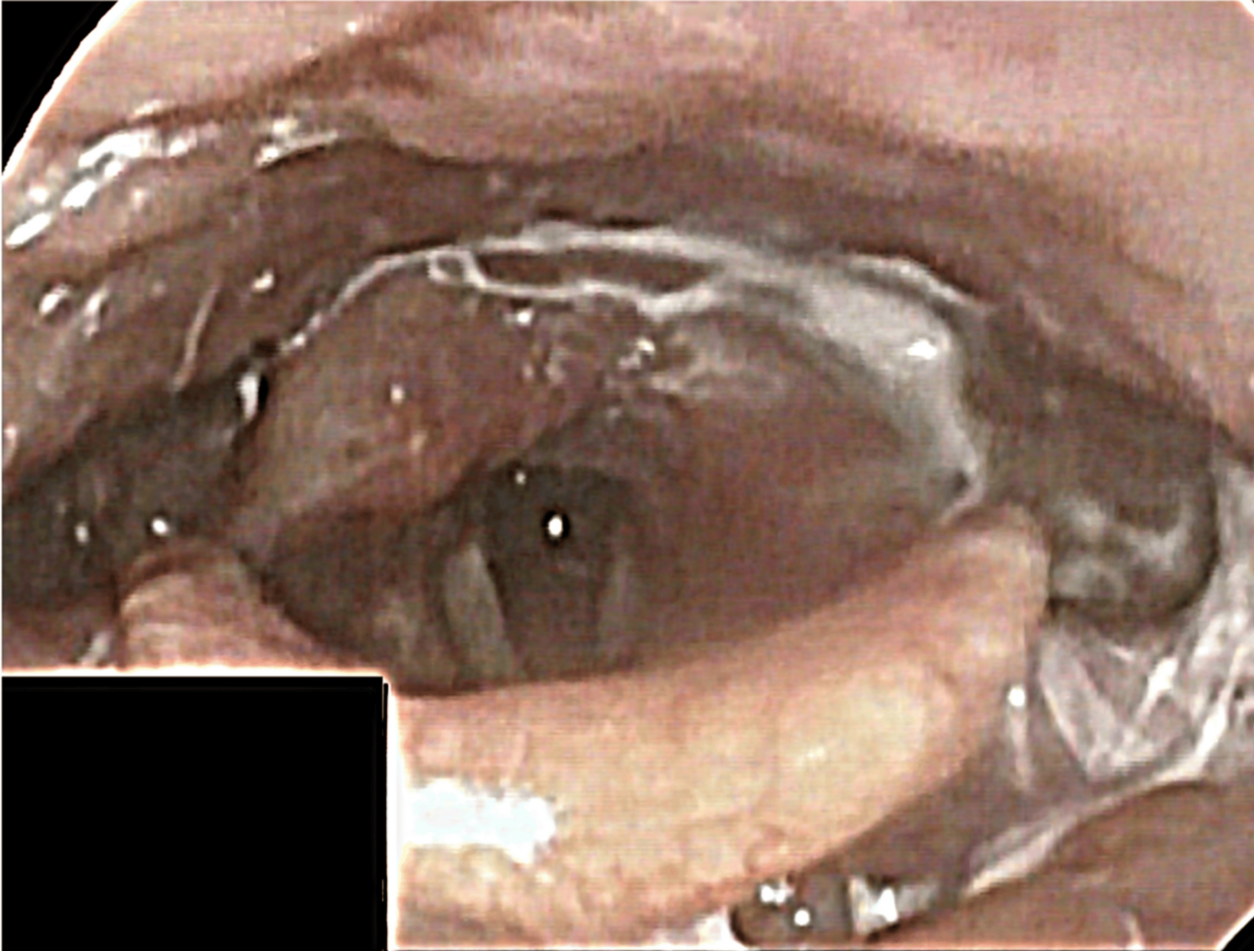
Laryngoscopy showing improvement of right vocal cord paralysis and bilateral edema of the arytenoid fold

After switching to the cuff-button-like cannula, the patient was able to speak. However, she had a hoarse voice. After one week, her hoarseness improved. On the 14th day of hospitalization, VE showed mild improvement in right vocal cord abduction paralysis, arytenoid edema, and opening of the right upper esophageal sphincter. However, VF indicated that the left upper esophageal sphincter still showed poor opening. A follow-up VE on the 23rd day after switching to the smaller-diameter nasogastric tube revealed complete improvement in the right vocal cord paralysis (Figure 3).

As the patient’s NGTS improved, her dysphagia also showed improvement. In addition, VF on the 23rd day of hospitalization revealed that the poor opening of the upper esophageal sphincter was improved. On the 56th day of hospitalization, the cuff-button-like cannula was removed, and the tracheostomy hole closed spontaneously. On the 77th day of hospitalization, the patient’s swallowing ability improved to a FILS level of 9, indicating no food restrictions and the ability to consume three meals orally. The patient was discharged on the 84th day of hospitalization.

This case study was conducted in accordance with the CARE guidelines for case reporting, and written informed consent was obtained from the patient.

## Discussion

We identified two important clinical insights. First, replacing the nasogastric tube with a smaller-diameter tube can improve NGTS symptoms. Second, NGTS can cause dysphagia, and its improvement may subsequently lead to improvement in dysphagia.

First, replacing the 12-Fr nasogastric tube with a smaller-diameter (8-Fr) tube may help improve NGTS. The proposed mechanism underlying NGTS likely involves tissue injury caused by continuous pressure on the narrow anatomical region between the hypopharynx and the cervical esophagus [8]. The standard treatment for NGTS typically involves immediate removal of the nasogastric tube to relieve this pressure. In fact, approximately 73% of reported NGTS cases have been managed by tube removal [2]. Several studies have also used antimicrobials, corticosteroids, and proton pump inhibitors to treat NGTS [9,10]. However, their effectiveness as established therapies remains unclear, and none of these drugs have been used in this case. Replacing a standard nasogastric tube with a softer, smaller-diameter tube has shown some promise in preventing NGTS [3]. However, considering that NGTS has been reported even with small-diameter tubes, the relationship between NGTS and nasogastric tube size remains unclear [1]. This is the first reported case demonstrating that NGTS can be a direct cause of dysphagia and that replacing the gastric tube with a smaller-diameter tube can improve NGTS and associated dysphagia. There have been reports of NGTS developing as late as three months after insertion of an 8-Fr nasogastric tube, likely due to tissue damage caused by the prolonged placement of even small-diameter nasogastric tubes [11]. Since NGTS is caused by sustained pressure from the nasogastric tube, a smaller and softer tube may reduce tissue damage. However, NGTS symptoms can become severe, potentially requiring tracheostomy or even resulting in death because of upper airway obstruction [2]. Therefore, in the absence of a tracheostomy and when there is a risk of upper airway obstruction, complete removal of the nasogastric tube remains the optimal treatment. In the present case, the patient had a strong gag reflex, making it difficult to perform intermittent tube feeding. Moreover, we aimed to avoid invasive central intravenous feeding because of a previous panic attack experienced at another hospital. Conversely, because the patient had a tracheostomy, she had no risk of upper airway obstruction from NGTS. Therefore, we opted to replace the nasogastric tube with a smaller-diameter tube instead of removing it. Twenty-three days after the diagnosis, we confirmed the healing of NGTS. Although the healing process may take longer, this case demonstrates that NGTS can be treated without removing the nasogastric tube when continued tube use is necessary. Therefore, removal of the nasogastric tube should be the primary priority in cases of NGTS with a risk of upper airway stricture. Conversely, replacing the tube with a smaller-diameter nasogastric tube may be a viable treatment option in patients with a tracheostomy and no risk of upper airway stricture.

Second, NGTS could be a contributing factor to dysphagia. Previous studies have found that patients with a nasogastric tube tend to have more residue in the pyriform sinuses and a reduced cough reflex [12,13]. The nasogastric tube may impair swallowing function during the pharyngeal phase. While approximately 11% of patients reportedly experienced dysphagia in a meta-summary of case reports [2], the severity and specific characteristics of dysphagia are not well documented. While there have been cases in which oral intake was compromised due to throat pain of NGTS [14], reports of dysphagia resulting from bilateral or unilateral edema of the arytenoid fold in NGTS are limited. VE often reveals bilateral or unilateral edema of the arytenoid fold and vocal cord paralysis in patients with NGTS [15,16]. Dysphagia may occur in patients with NGTS because boluses cannot easily pass through the pharyngo-esophageal segment and can flow into the airway by overcoming the aryepiglottic folds through the narrowing of the piriform recess because of arytenoid fold edema. In patients with dysphagia, nasogastric tubes are often inserted to support nutritional intake when oral intake is difficult. However, the nasogastric tubes may contribute to NGTS development and exacerbate dysphagia during rehabilitation. In the present case, the patient’s dysphagia gradually resolved as her NGTS improved, enabling her to take three meals orally after rehabilitation and tracheostomy decannulation.

In the present case, NGTS was diagnosed by VE. Some patients admitted for dysphagia rehabilitation have a tracheostomy or nasogastric tube. When a tracheostomy is present, NGTS may remain undiagnosed because upper airway obstruction caused by NGTS does not lead to respiratory distress or inspiratory wheezing in these patients. Consequently, NGTS treatment may be delayed, potentially prolonging dysphagia if NGTS is not considered as a cause. Since this patient had undergone a tracheotomy, there was no wheezing, which is a common symptom of NGTS, and the only symptoms were pharyngeal discomfort and dysphagia. In such cases, NGTS may be detected late. Therefore, it is essential to proactively assess for NGTS by evaluating vocal cord movement and arytenoid fold edema through VE examinations. Given that nasogastric tubes are frequently used in dysphagia rehabilitation, this complication should be closely monitored in patients with a nasogastric tube. It is important to use VE to check for the development of NGTS in patients with dysphagia while a nasogastric tube is in place, even if the patient has the typical symptoms of NGTS.

## Conclusions

Replacing the 12-Fr nasogastric tube with a smaller-diameter (8-Fr) tube can improve NGTS and associated dysphagia. It offers a feasible option for patients requiring continued nasogastric tube feeding without removing the tube. NGTS should be considered in patients with a nasogastric tube who present with dysphagia.
